# Salivary total α-synuclein, oligomeric α-synuclein and *SNCA* variants in Parkinson’s disease patients

**DOI:** 10.1038/srep28143

**Published:** 2016-06-23

**Authors:** Wenyan Kang, Wei Chen, Qiong Yang, Lina Zhang, Linyuan Zhang, Xiaoying Wang, Fangyi Dong, Yang Zhao, Shuai Chen, Thomas J. Quinn, Jing Zhang, Shengdi Chen, Jun Liu

**Affiliations:** 1 Department of Neurology & Institute of Neurology, Ruijin Hospital affiliated to Shanghai Jiaotong University School of Medicine, Shanghai, 200025, China; 2 Department of Neurology, Ruijin Hospital North affiliated to Shanghai Jiaotong University School of Medicine, Shanghai, 201801, China; 3 Department of Biostatistics, Shanghai Jiaotong University School of Medicine, Shanghai, 200025, China; 4 Department of Radiation Oncology, Albert Einstein College of Medicine of Yeshiva University, NY 10461, USA; 5 Department of Pathology, University of Washington School of Medicine, Seattle, WA 98107, USA

## Abstract

The present study was to evaluate the diagnostic value of salivary total and oligomeric α-synuclein levels in PD. Furthermore, we sought to explore the relationship between salivary total α-synuclein and α-synuclein *SNP* variants levels. 201 PD patients and 67 controls were recruited, of which there also had the genetic information of two positive α-synuclein (SNCA) loci. Salivary total α-synuclein was assayed using a highly sensitive Luminex assay and oligomeric α-synuclein was quantified by the combination of Gel filtration chromatography and Western blot, respectively. From our analysis,No difference in salivary total α-synuclein levels was found between PD patients and healthy controls, it decreased with age in PD patients, and was closely associated with genotypic distribution of rs11931074 and rs894278 in PD, respectively. After controlled for age and genders, G allele of rs11931074 was correlated with lower salivary total α-synuclein levels, while G allele of rs894278 was also correlated with the higher levels. Simultaneously, the further study was shown that salivary oligomeric α-synuclein in PD patients significantly increased comparing to healthy controls. In conclusions,our study firstly demonstrated that salivary total α-synuclein levels could be manipulated by different α-synuclein SNPs and salivary oligomeric α-synuclein could be a potential diagnostic indicator of PD.

Parkinson’s disease (PD), the second most common neurodegenerative disease, is difficult to diagnose earlier due to its insidious onset. In addition to the vast clinical heterogeneity and complexity of PD, there are currently no suitable biochemical markers that reflect disease phenotype or progression.

α-synuclein has been intensely investigated as a plausible PD biomarker due to the fact that it is a major component of Lewy bodies, the pathological hallmark of PD^1^. In the past few years, accumulating evidence demonstrated that α-synuclein could be detected in a series of biofluids, such as cerebrospinal fluid (CSF)^2^, serum/plasma^3^ and saliva^4^. However, studies focusing on CSF and serum α-synuclein yielded conflicting results, which was in part due to disparities in methods, antibodies used and control of confounding factors such as red blood cell and platelet contamination. There is substantial evidence to suggest that the conversion of α-synuclein from soluble monomers to aggregated, insoluble forms in the brain is a key event in the pathogenesis of PD and related diseases. Some studies reported that CSF and plasma samples from patients with PD contain high levels of α-synuclein oligomers^5^,^6^ and the RBC α-synuclein oligomer/total protein ratio can be a potential diagnostic biomarker for PD^7^. Pathological studies showed that α-synuclein could also be found in the submandibular gland of PD patients^8^, which is the primary source of human salivary volume. Saliva is an attractive biofluid by virtue of its accessibility and homogeneity without concern of blood contamination. Indeed, in our early study utilizing a small sample size, salivary α-synuclein seems to be a novel potential biomarker for PD^4^. However, the actual diagnostic value of salivary α-synuclein and relevant phenotypic and genotypic characteristics were not thoroughly investigated. Furthermore, the levels of salivary α-synuclein might not only be dependent on disease status but might also be modified by genetic variability, i.e., in the α-synuclein (SNCA) gene itself.

Mutations or multiplications in the SNCA gene cause a rare familial PD^9^. In addition, susceptibility variants in Chinese patients have been identified in our previous study, which genome-wide association studies (GWAS) and validation studies have identified multiple variants in different regions of SNCA that were associated with susceptibility of sporadic PD^10^,^11^. Altered expression of α-synuclein is considered one of the potential mechanisms contributing to the association between SNCA variants and PD development, indicating the possible correlation between genetic and biological markers. Previous report suggested that the variant tagged by rs356219 might regulate α-synuclein expression in a dose-dependent manner^12^,^13^. Whether a correlation exists between common risk-associated SNCA polymorphisms and peripheral α-synuclein levels such as saliva is not yet known.

## Materials and Methods

### Participants

From March 2011 to December 2012, 201 PD patients, who fulfilled the UK PD brain bank criteria^14^, were recruited from the movement disorders clinic at the Department of Neurology, Ruijin Hospital affiliated to Shanghai Jiaotong University School of Medicine, Shanghai, China. None of the patients had undergone functional neurosurgery for PD. 67 healthy controls were recruited within the local community of the Luwan district in Shanghai. Demographic information is listed in Table 1 for all patients/subjects. Written consent forms were obtained from all participants. The study was approved by the Ethics Committee of Ruijin Hospital affiliated to Shanghai Jiaotong University School of Medicine, Shanghai, China and the methods were carried out in accordance with the approved guidelines. The informed consent was obtained from all subjects.

### Clinical profiling

For PD patients, disease stage was determined using Hoehn and Yahr staging system. The motor subscale of Unified Parkinson’s Disease Rating Scale (UPDRS III) was used to evaluate motor function^15^.

### Genotyping of rs894278 and rs11931074

Genetic information of recruited PD patents was obtained. Briefly, Genomic DNA was extracted from peripheral blood through a standardized phenol/chloroform extraction method. SNCA single nucleotide polymorphisms (SNPs) (rs894278, rs11931074) were genotyped by direct sequencing. The regions of SNCA containing rs894278 and rs11931074 were amplified by polymerase chain reaction (PCR) separately in a total volume of 20 μl. Genotypes of the two loci were determined by direct DNA sequencing performed on a 3730xl DNA analyzer (Applied Biosystems, Foster City, CA, USA).

### Saliva sample collection and Luminex assay

All saliva samples were collected from patients with PD (n = 201), as well as healthy controls (n = 67) as described previously^16^. Briefly, saliva was collected in a resting and unstimulated state (no food, chewing gum, etc). All collections were performed between 9:00 am and 11:00 am to control for any potential confounding effects of circadian rhythm. After collection, the whole sample was immediately placed on ice, and then Protease Inhibitor Cocktail (100 μl/1 ml of whole saliva, Cat#P2714, Sigma Aldrich, St. Louis, MO, USA) was added to the sample to minimize protein degradation, and the sample was vortexed repeatedly followed by centrifugation first at a low spin of 2,600 × g for 15 inutes then at a high spin of 15,000 × g for 15 minutes at 4 °C to remove particles. The supernatant was then divided into 0.5 ml aliquots, and then stored at −80 °C before analysis. Salivary total protein concentration was measured using a BCA protein assay kit (Pierce, Rockford, IL, USA).

Magnetic COOH beads (Cat#MC10052-01, Bio-Rad, USA) were chemically coupled with a mouse (monoclonal) anti-human α-synuclein antibody (Cat#AHB0261, Invitrogen, USA) with the amine coupling kit (Cat#171-406001, Bio-Rad, USA) according to the manufacture’s protocol. Briefly, 100 μl beads were activated with 10 μl EDC (1-ethyl-3-[3-dimethylaminopropyl] carbodiimide, 50 mg/ml) and Sulfo-NHS (N-hydroxysulfosuccinimide, 50 mg/ml) in the ProteOn™ Amine Coupling Kit (Cat#1782410,Bio-Rad,USA).20 μg of primary antibody was added to the activated beads and incubated for 2 hours. The coupled beads were re-suspended in 150 μl of storage buffer or an alternate storage buffer to complement the protein assay. Determination of bead concentration was performed using a Coulter Z2 counter or a hemocytometer to validate the efficiency of the coupling reaction. The coupled beads were stored at 4 °C and covered with aluminum foil.

### Magnetic bead-based luminex assay

Salivary α-synuclein levels were measured using established Luminex assays as described previously^16^,^17^. Capturing antibody-coupled beads (about 2500 beads per well) were first added to 96 well Bio-Plex Pro Flat Bottom Plates (Cat#171025001, Bio-Rad, USA) and washed twice with washing stations using the reagent kit (Cat#171304071, Bio-Rad, USA). Then, 50 μl diluted recombinant human α-synuclein (Prospec) was used as standards. Saliva samples were diluted in equal sample volume. Next, standards, samples, and sample blanks were loaded on the magnetic plate with incubation for 2 hours × 1000 rpm on a plate shaker at room temperature in the dark, and then the plate was washed 3 times. Subsequently, detecting antibodies (2 μg/ml, biotinylated anti-human α-synuclein antibody, R&D systems, Minneapolis, MN, USA) were added at 50 μl per well, and the plates placed on a rotator at room temperature for 60 minutes, followed by washing 3 times, and the streptavidin-PE was diluted with assay buffer in the reagent kit for 30 minutes. The plate was then washed for 3 times and re-suspended in 125 μl assay buffer with shaking for 3 minutes, and then read via a Liquichip Luminex 200TM. The concentration of samples was calculated by comparison to a best-fit standard curve using a sigmoidal 5-parameter logistic equation. The salivary α-synuclein signal-to-background ratio was 68. The recovery rate was close to 87% and coefficient of variation (CV) in duplicate was less than 15%. Finally, the Luminex assay demonstrated low day-to-day as well as plate-to-plate signal variability (<10%), with high signal reproducibility.

### Size exclusion chromatography

Equal amount of saliva from participants (5 samples in each group) were pooled together to 1 ml, and then concentrated to 0.5 ml in order to increase the protein concentration by a low temperature vacuum drying apparatus (Millrock Technology, USA). 0.2 ml Pooled saliva sample were then analyzed by high-performance liquid chromatography on a Superdex75 (GE health care, USA) column that had been equilibrated with a solution containing 50 Mm NH_4_HCO3 (pH = 7.4). Proteins were eluted from the column with the equilibration solution at a flow rate of 0.8 ml/min. Fractions of 0.5 ml were collected and concentrated by centrifugation under vacuum.

### Western Blotting

Salivary proteins were eluted from the column with the equilibration solution, and the concentrated fractions were boiled in 4 × SDS loading buffer, followed by the samples being separated by SDS–polyacrylamide gel electrophoresis (SDS-PAGE), and transferred to a polyvinylidene fluoride (PVDF) membrane and detected by immunoblotting analysis. Band intensities were quantified by densitometry analyses using ImageJ software. All of the experiments were performed at least 3 times.

### Statistical analysis

Statistical analyses were performed using SAS version 9.2 (SAS Institute, Cary, NC, USA) and GraphPad Prism for Windows version 5.0 (GraphPad Software, San Diego, CA, USA). Paired or unpaired Student’s t-test was used for statistical analyses. To compare enumeration data among groups, Chi-square test or Fisher’s exact test was used. We analyzed the data by *t* test or one-way analysis of variance (ANOVA) if the data was normally distributed. Non-parametric Kruskal–Wallis ANOVA and Wilcoxon rank sum test was used, followed by a Mann–Whitney U-test for continuous variables if data was not normally distributed. Additionally, to determine whether a relationship among variables was present, we used an analysis of covariance (ANCOVA) model after rank transformation of the non-normal data to control for potential contributions secondary to outliers. The analyses were done with and without adjustment for potential confounding of the baseline variables-i.e. age, gender. For the measurement data, to determine whether a relationship among variables was present, we used the non-parametric spearman rank correlation analysis. The Cochran-Mantel-Haenszel test was applied to analyze the enumeration data. All values were expressed as mean ± SEM or mean ± SD.A two-tailed p value < 0.05 was defined as a statistically significant difference.

## Results

### The characteristics of salivary total α-synuclein in PD groups

**Figure 1:**
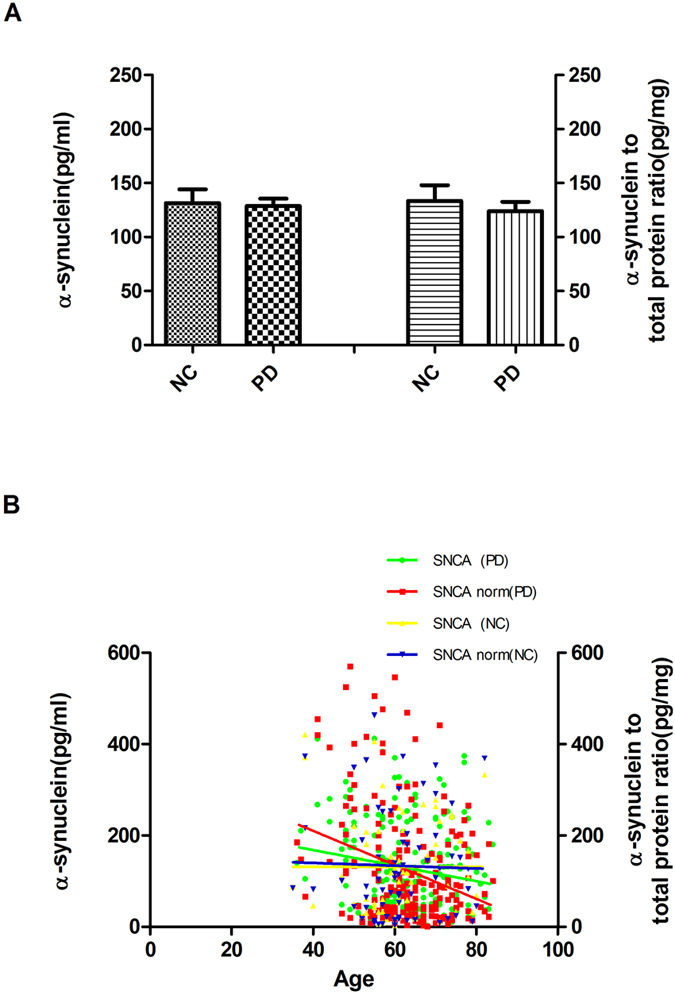
The characteristics of total salivary α-synuclein in PD group. **(A)** Salivary α-synuclein levels were measured in individual normal controls (NC) and PD samples by Luminex. Quantitative Luminex analysis of salivary α-synuclein levels in patients with PD and NC, before and after controlling for potential contributions secondary to outliers (e.g. age, gender) by the nonparametric Mann-Whitney U test (for pair wise comparisons) and the covariance (ANCOVA) model analysis. There was no significant difference in mean salivary α-synuclein levels between cases and controls (*p* = 0.97), also no significant differences were found when normalized (*p* = 0.49). **(B)** Using linear regression analysis with spearman’s correlation, levels of α-synuclein in saliva tended to decrease with age in PD group (r = −0.16 *p* = 0.02, r = −0.21, *p* = 0.003 when normalized) but not in controls (*p* > 0.05; *p* > 0.05 when normalized). Error bars represent mean ± SEM.

To compare the difference of salivary α-synuclein level between PD group and health control, 201 PD patients and 67 health control were recruited. The demographic data was listed in Table 1. There was no significant difference in mean saliva α-synuclein levels between patients and controls (128.66 ± 98.21 vs. 131.31 ± 104.21 ng/ml; *p* = 0.97), though no statistically significant difference was found when protein concentrations were normalized (123.83 ± 124.91 vs. 133.52 ± 118.20 pg/mg, *p* = 0.49, Fig. 1A). When we compared the levels of salivary α-synuclein between males and females whether in PD or control groups, no obvious difference was found (*p* > 0.05, *p* > 0.05 when normalized Fig. S1A). In addition, in terms of disease phenotype, salivary α-synuclein levels did not correlate with either H-Y stage or UPDRS-III score (*p* > 0.05, Fig. S1B,C). Finally, no correlation was found between therapeutic use,disease duration and total α-synuclein levels, whether normalized or not (*p* > 0.05, Fig. S1D,E). For further investigating the characteristics of salivary total α-synuclein in different groups, bivariate correlation analysis revealed that salivary α-synuclein levels decreased with age in PD patients (r = −0.162, *p* = 0.021; r = −0.211, *p* = 0.003 when normalized, Fig. 1B), but not in healthy controls (*p* = 0.32, *p* = 0.74 when normalized, Fig. 1B). The above results indicated that salivary α-synuclein levels were mainly affected by age in PD patients, but not by gender, disease duration or pharmacotherapy.

### Salivary total α-synuclein associated with SNCA subsets of PD

**Figure 2:**
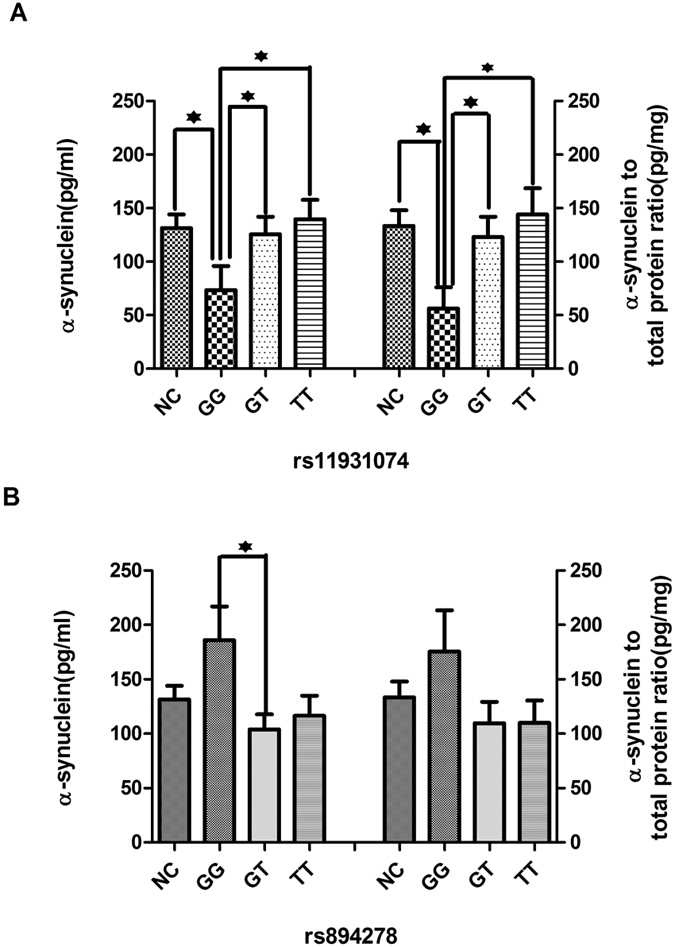
Association between total salivary α-synuclein and two SNCA variants in PD. (**A**) After controlling for potential contributions secondary to outliers (e.g. age, gender), α-synuclein levels in saliva were associated with genotype distribution of rs11931074. The levels in the GG group were significantly lower than those in the GT (*p* = 0.033, *p* = 0.022 when normalized), TT (*p* = 0.038, *p* = 0.027 when normalized). **(B)** The levels in the rs894278 GG group were higher than those in the GT (*p* = 0.023, *p* = 0.089 when normalized), TT (*p* = 0.052, *p* = 0.11 when normalized). Error bars represent mean ± SEM.

Since previous studies have been shown that there exists the correlation between genetic and PD development^12^,^13^, we sought to perform subgroup analysis to ascertain the relationship of salivary total α-synuclein levels to genetic and phenotypic presentation. According to SNCA SNP results (rs894278 and rs11931074) in our previous GWAS study in China^10^, recruited PD patients were divided into different groups, of which salivary α-synuclein were analyzed. The results were firstly shown that salivary α-synuclein was associated with genotype distribution of SNCA rs894278 and rs11931074. After controlling for age, the level of salivary α-synuclein in the rs11931074 GG group was significantly lower than those in the GT, TT group (*p* = 0.033, *p* = 0.038, Fig. 2A), whether normalized or not (*p* = 0.022, p = 0.027, respectively, Fig. 2A). On the other hand, the level of salivary α-synuclein in the GG group was higher than those in the GT, TT groups (*p* = 0.023, *p* = 0.051, Fig. 2B), but not the α-synuclein to total protein ratios (*p* = 0.11, *p* = 0.089, respectively, Fig. 2B).

### The characteristics of salivary oligomeric α-synuclein in PD patients

**Figure 3:**
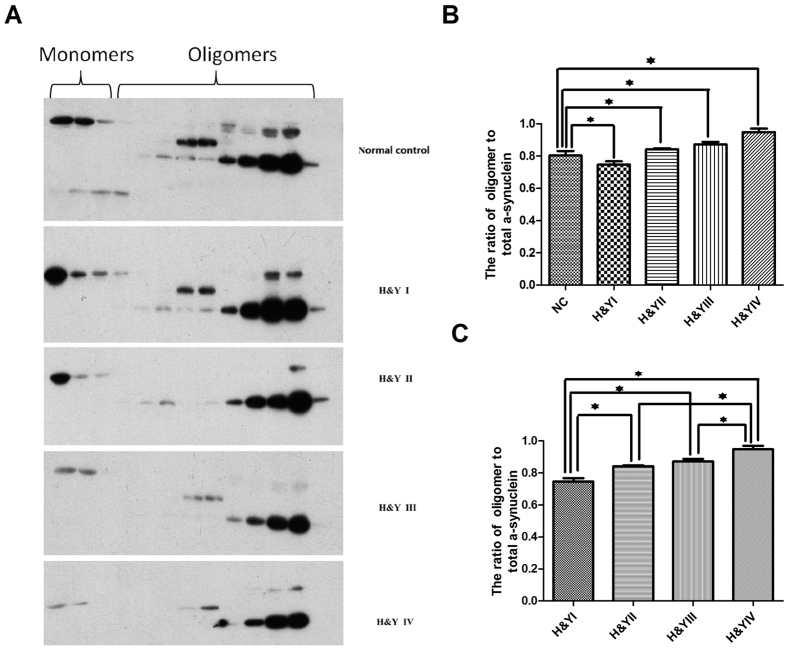
The diagnostic value of salivary oligomeric α-synuclein in PD. Salivary proteins were analyzed by high-performance liquid chromatography on a Superdex75 (GE Health Care, USA) column that had been equilibrated with a solution containing 50 mM NH_4_HCO_3_ (pH = 7.4). Proteins were eluted from the column with the equilibration solution and the resulting fractions were subjected to immunoblotting analysis with antibodies to α-synuclein. All the gels have been run under the same experimental conditions and the representative cropped gels were shown in the figure.The positions of monomers, oligomers of α-synuclein are shown (Fig. 3A). From the analysis by Image J, the ratio of oligomer/total α-synuclein decreased significantly in H&Y stage I (*p* = 0.001) and increased in H&Y II-IV stages compared to normal controls (*p* = 0.037, *p* = 0.002, *p* = 0.000, respectively, Fig. 3B). The results also revealed significant differences at each H&Y stage (74.7%, 84.2%, 87.2%, 94.8%, respectively,* p* = 0.000, Fig. 3C). An analysis of covariance (ANCOVA) model was used.Error bars represent mean ± SD from 3 independent experiments.

Previous studies have been shown that oligomeric α-synuclein of CSF or plasma increased in PD patients comparing to healthy controls^5^,^6^. Though we didn’t find any difference in salivary total α-synuclein between PD patients and healthy controls, we went ahead to investigate whether salivary oligomeric α-synuclein could be changed in PD patients. We have separated oligomeric “soluble aggregates” from monomeric forms in saliva through Gel filtration chromategraphy followed by immunoblot analysis with antibodies to α-synuclein (Fig. 3A). Recruited PD patients were divided into different groups according to Hoehn & Yahr (H&Y) disease stages. Our results were shown that the ratio of oligomer/total α-synuclein decreased significantly in H&Y stage I (*p* = 0.001) and increased highly in H&Y II-IV stages (*p* = 0.037, *p* = 0.002, *p* = 0.000, respectively, Fig. 3B) compared to normal controls, which the maximum ratio of oligomeric α-synuclein appeared in the late stage of PD patients. To determine whether salivary oligomeric α-synuclein could serve as a potential biomarker of PD progression, we compared the amount of oligomeric α-synuclein in total α-synuclein at each stage of PD patients. The results revealed significant differences at each H&Y stage (74.7%, 84.2%, 87.2%, 94.8%, respectively, *p* = 0.000, Fig. 3C), which suggested that salivary oligomeric α-synuclein might be a potential biomarker for disease progression monitoring of PD patients.

## Discussion

The present study demonstrated that: (1) Besides characterization of the nature of α-synuclein levels in saliva from PD patients, no difference of total salivary α-synuclein levels was found between PD and healthy controls. However, there was some association between total salivary α-synuclein and age, though no obvious association was found between its levels with disease stages, motor symptoms. (2) Besides age, genotypic distribution of rs894278 and rs11931074 may influence the level of total salivary α-synuclein in PD patients. (3) Our results indicated that salivary samples from patients with PD contain high levels of α-synuclein oligomers and could be a potential biomarker for diagnosis and monitoring disease progression of PD.

The optimal diagnostic biomarker would be of tremendous importance for diagnosis and clinical trials of PD. Furthermore, a progression marker would be important to monitor disease progression for upcoming clinical trials with putative neuroprotective agents objectively. The discovery of α-synuclein as a major component of Lewy bodies (LBs), the neuropathological hallmark of PD initiated the investigation of α-synuclein as a biomarker. A number of studies have assayed the alterations in α-synuclein levels in CSF and plasma to evaluate the potential value as a diagnostic biomarker for PD, however, the previous studies has led to discrepant findings in several studies^18^,^19^. These conflicting results may be due to different sample processing, different used assay design and other factors could be recognized that affect the assay signal and cause variation in measurements. Besides the use of different antibodies with different affinities, recognizing different α-synuclein species; There is no standardization of the standard peptides used in the various assays; Blood contamination would be another identified confounding factors, due to traumatic lumbar puncture, has been recognized as a serious problem for α-synuclein quantization in CSF. The levels of α-synuclein in serum and plasma are up to 10,000-fold higher than in CSF as given above; Red blood cells (RBCs) contain high levels of α-synuclein and detection of plasma/serum α-synuclein couldn’t avoid contamination arising from hemolysis^20^,^21^.

Because the distribution of α-synuclein pathology points toward PD as a systemic disease with the affection of the peripheral nervous system and organs, there may be chances to detect a peripheral marker in more easily accessible biological fluids than CSF. α-synuclein has also been quantified in saliva, could be useful in detecting PD and is more readily accessible compared to other biofluids. The sources of salivary α-synuclein are currently unknown. Neuropathological studies have shown that α-synuclein had been visualized in multiple tissues include the superior and inferior salivary nuclei parasympathetic salivary ganglia, salivary gland and vagus nerve^19^. α-synuclein may spread from neuronal cell bodies of salivary neurons, along axons, to the synaptic terminals around the epithelial cells of salivary glands,where it also accumulates in the saliva^22^, the nerves innervated salivary glands release α-synuclein into the saliva by yet-to-be defined mechanisms,a fraction of this secreted protein may has been linked to exosomes, membrane vesicles of endocytic origin^4^,^19^,^23^. Previous studies have demonstrated that exosomes are present in human whole saliva^24^.The whole saliva contained at least two types of exosomes (exosome I and exosome II) that are different in size and protein composition, 101 and 154 proteins were identified from exosome I and exosome II, respectively, by Proteomic analysis. Emerging evidence suggests that aggregated α-synuclein spreads from cell-to-cell in a prion-like manner, resulting in transmission of aggregation and neurodegeneration, and secretion of α-synuclein via exosomes has been proposed to amplify and propagate PD pathology^25^. CSF α-synuclein can be readily transported into blood, plasma exosomal α-synuclein is likely CNS-derived and increased in Parkinson’s disease^23^. The relationship of α-synuclein levels in saliva, blood, and cerebrospinal fluid need further exploration. Though we found no association was found between levels of α-synuclein in salivary and plasma(Suppl. Fig. 2, p > 0.05).

The correlation between salivary α-synuclein and the presence or severity of PD remains controversial. Some studies showed no significant differences were observed for either the cellular component, supernatant or cellular pellet lysate by Luminex assays^4^,^26^. However, other studies with a slightly small cohort α-synuclein levels significantly decreased in PD patients compared to controls using ELISA^22^,^27^. Besides taking into consideration methods of analysis(for example ,different detected antibodies, sample contamination control),in order to adequately assessing salivary α-synuclein levels, much larger cohorts of PD patients and healthy controls may be beneficial. In our study 201 PD patients and 67 controls were recruited, analysed by Luminex assays. Though there was a slight trend for α-synuclein to decrease in patients with Parkinson’s disease compared to controls ,we showed total salivary α-synuclein levels would not suffice as a single biomarker for diagnosis. We didn’t find association between total salivary α-synuclein levels with disease duration, H&Y stage or UPDRS-III, either. Because the clinical measures of disease severity used, i.e. H&Y stage and UPDRS-III, may have low sensitivity for small differences in disease severity,and the results were similar to the recent works in the CSF^28^.

We found that salivary α-synuclein levels decreased with age in PD patients, but not in healthy controls.while α-synuclein levels in the CSF were significantly increased as a function of age, especially in normal controls^2^. Another study revealed that α-synuclein levels in blood plasma from healthy individuals markedly decrease between the 3rd and 5th decade of life^29^. Salivary α-synuclein may not only be a potiential marker for diagnosis but also reflect biological age and qualify as a candidate biomarker of aging to advance life expectancy. Based on evidence that protein aggregation constitutes an inherent phenomenon of aging, imbalances of α-synuclein levels increases with age.As an aggregation-prone and amyloid-forming protein,it is reasonable to assume that the detecting epitope can be conformationally masked in aggregates, which then remain undetected in the assay,which may partly explain the decreased trend of α-synuclein levels with age in PD patients.

To our surprise, our study found that besides age, minor allele G of rs894278 was associated with increased salivary α-synuclein levels in patients with PD via an additive model, while minor allele G of rs11931074 was associated with decreased salivary α-synuclein levels. Polymorphisms in non-coding regions of SNCA have also been shown to contribute to the risk of sporadic PD. As introns of the SNCA gene, SNP rs894278 and rs11931074 confer risk and protective effect for PD as demonstrated by our recent validation study with a large sample size respectively. SNCA rs894278 in PD patients with early onset indicated significant differences in allele frequency compared with controls. However, in a subgroup analysis of patients with a positive family history for the SNPs, there were no differences compared with controls whereas the minor allele G of rs11931074 reduces the risk of PD progression^10^. The present study suggested that the intron *SNCA* rs894278 /rs11931074 may also have biological ramifications, the mechanism of which warrants further research. Our finding is consistent with the results of previous study which revealed that the risk-associated C allele of rs356219 was also correlated with higher transformed plasma α-synuclein levels in cases under an adjusted additive model^12^. Ethnic effects also play an important role in genetic susceptibility of PD, which further multi-center validation studies are warranted to determine the diagnostic value of salivary α-synuclein and its components for PD in different populations.

Oligomeric forms of α-synuclein have been found to be elevated in the CSF of PD patients as compared with controls. As a result, when only oligomers are measured, the sensitivity and specificity were found to be 75.0% and 87.5%, respectively, which increases to 89.3% and 90.6% when the ratio of oligomers/total α-synuclein is calculated^30^. Similar alterations have also been observed in saliva^22^ and plasma^5^. Consequently, the origin of α-synuclein and the diagnostic value of different components of α-synuclein in saliva merit further investigation. Sephadex G75 columns have been used successfully to detect soluble oligomers of α-synuclein in extracts from PD and DLB brains as well as in plasma from PD patients^5^. In the present study, we also measured salivary α-synuclein oligomers through Gel filtration chromatography followed by immunoblot analysis. The results revealed that salivary α-synuclein might be a potential biomarker for diagnosis and monitoring disease progression of PD. The present results are consistent with previous findings showing an elevation of oligomeric α-synuclein in plasma, CSF,saliva and RBCs in PD as compared to non-PD patients, supporting that increased α-synuclein oligomer in plasma and CSF as potential diagnostic biomarker for PD^5^,^6^,^7^,^22^. Exosomes may be involved in the potential mechanism of aggregation of α-synuclein.Grey M’s research showed that aggregation of exogenous α-synuclein is accelerated by exosomes and the lipid composition of the exosomes is crucial for the aggregation process^25^. Another study suggested that CSF exosomes from patients with dementia with Lewy bodies and Parkinson’s disease induce α-synuclein oligomerization^31^.Because saliva can avoid contamination arising from hemolysis, the present method should be more stable and reproducible compared with those detecting α-synuclein oligomers in plasma and CSF. Exosomal α-synuclein as a potential future biomarker in α-synuclein-related neurodegenerative disorders^31^. Further investigations are needed to explore whether the exosomal α-synuclein as a potential future biomarker in α-synuclein-related neurodegene rative disorders.

In summary, we found that oligomeric forms of α-synuclein could be a potential biomarker for diagnosis and monitoring disease progression of PD patients and salivary total α-synuclein could be useful to screen different phenotype or genotype of PD patients, which the level is inversely associated with age, and closely correlated with genotypic distribution of rs894278/rs11931074 in PD.

## Additional Information

**How to cite this article**: Kang, W. *et al*. Salivary total α-synuclein, oligomeric α-synuclein and *SNCA* variants in Parkinson’s disease patients. *Sci. Rep.* **6**, 28143; doi: 10.1038/srep28143 (2016).

## Supplementary Material

Supplementary Information

## Figures and Tables

**Table 1:** Demographic, clinical and biological data in PD and controls.

	Control	PD
Number of cases	67	201
Sex (M/F)	41/26	122/79
Age (years)	61.04 ± 10.01	63.18 ± 9.67
Cases of drug treatment		133:8:22:38^a^
Salivary α-synuclein (pg/ml)	131.31 ± 104.21	128.66 ± 98.21
Normalized salivary α-synuclein (pg/mg)	133.52 ± 118.20	123.83 ± 124.91

^a^Number of patients with Parkinson’s disease who were treated with carbidopa/levodopa alone or together with other anti-parkinsonism drugs (**Type I**) versus those treated with dopamine agonists but not levodopa (**Type II**) versus those treated with other anti-Parkinson’s disease medications (e.g. monoamine oxidase B inhibitors and amantadine) only (**Type III**) versus those not treated with any anti-parkinsonism drugs (no Parkinson’s disease medication) when the saliva samples were obtained (**Type IV**).

## References

[b1] Halliday G., Lees A. & Stern M. Milestones in Parkinson’s disease–clinical and pathologic features. Mov Disord. 26(6), 1015–21 (2011). 21626546 10.1002/mds.23669

[b2] Hong Z. . DJ-1 and alpha-synuclein in human cerebrospinal fluid as biomarkers of Parkinson’s disease. Brain. 133 (Pt 3), 713–26 (2010). 20157014 10.1093/brain/awq008 PMC2842513

[b3] Shi M. . Significance and confounders of peripheral DJ-1 and alpha-synuclein in Parkinson’s disease. Neurosci Lett. 9 480(1), 78–82 (2010). 20540987 10.1016/j.neulet.2010.06.009 PMC2943649

[b4] Devic I. . Salivary α-synuclein and DJ-1: potential biomarkers for Parkinson’s disease. Brain. 134 (Pt 7), e17 8 (2011). 10.1093/brain/awr015 PMC3122368 21349902

[b5] El-Agnaf O. M. . Detection of oligomeric forms of alpha-synuclein protein in human plasma as a potential biomarker for Parkinson’s disease. FASEB J. 20(3), 419–25 (2006). 16507759 10.1096/fj.03-1449com

[b6] Park M. J. . Elevated levels of α-synuclein oligomer in the cerebrospinal fluid of drug-naïve patients with Parkinson’s disease. J Clin Neurol. 7(4), 215–22 (2011). 22259618 10.3988/jcn.2011.7.4.215 PMC3259496

[b7] Wang X., Yu S., Li F. & Feng T. Detection of α-synuclein oligomers in red blood cells as a potential biomarker of Parkinson’s disease. Neurosci Lett. 10(599), 115–9 (2015). 10.1016/j.neulet.2015.05.030 25998655

[b8] Del Tredici K., Hawkes. C. H., Ghebremedhin E. & Braak H. Lewy pathology in the submandibular gland of individuals with incidental Lewy body disease and sporadic Parkinson’s disease. Acta Neuro pathol. 119(6), 703–13 (2010) 10.1007/s00401-010-0665-2 20229352

[b9] Narhi L. . Both familial Parkinson’s disease mutations accelerate alpha-synuclein aggregation. J Biol Chem. 2 274(14), 9843–6 (1999). 10092675 10.1074/jbc.274.14.9843

[b10] Liu J. . Analysis of genome-wide association syudy-linked loci in Parkinson’s disease of Mainland China. Mov Disord. 28(13), 1892–5 (2013). 23853107 10.1002/mds.25599

[b11] Simon-Sanchez J. . Genome-wide association study reveals genetic risk underlying Parkinson’s disease. Nat Genet. 41, 1308–1312 (2009). 19915575 10.1038/ng.487 PMC2787725

[b12] Mata I. F. . SNCA variant associated with Parkinson disease and plasma alpha-synuclein. Arch Neurol. 67(11), 1350–6 (2010). 21060011 10.1001/archneurol.2010.279 PMC3010848

[b13] Fuchs J. . Genetic variability in the SNCA gene influences alpha-synuclein levels in the blood and brain. FASEB J. 22(5), 1327–34 (2008). 18162487 10.1096/fj.07-9348com

[b14] Hughes A. J., Daniel S. E., Kilford L. & Lees A. J. Accuracy of clinical diagnosis of idiopathic Parkinson’s disease: a clinico-pathological study of 100 cases. J Neurol Neurosurg Psychiatry. 55(3), 181–4 (1992). 1564476 10.1136/jnnp.55.3.181 PMC1014720

[b15] Richards M., Marder K., Cote L. & Mayeux R. Interrater reliability of the Unified Parkinson’s Disease Rating Scale motor examination. Mov Disord. 9(1), 89–91 (1994). 8139610 10.1002/mds.870090114

[b16] Kang W. Y. . Salivary DJ-1 could be an indicator of Parkinson’s disease progression. Front Aging Neurosci. 6(6), 102 (2014). 24936184 10.3389/fnagi.2014.00102 PMC4047660

[b17] Wang X. Y. . Using gastrocnemius sEMG and plasma α-synuclein for the prediction of freezing of gait in Parkinson’s patients. PLos One. **9(2)**, 27, e89353 (2014). 24586710 10.1371/journal.pone.0089353 PMC3937335

[b18] Simonsen A. H. . The utility of α-synuclein as biofluid marker in neurodegenerative diseases: a systematic review of the literature. Biomark Med. 10(1), 19–34 (2016). 26314196 10.2217/BMM.14.105

[b19] Atik A., Stewart T. & Zhang J. Alpha-synuclein as a biomarker for Parkinson’s disease. Brain Pathol. 26(3), 410–8 (2016). 26940058 10.1111/bpa.12370 PMC6245641

[b20] Mollenhauer B. . Quantification of α-synuclein in cerebrospinal fluid as a biomarker candidate: review of the literature and considerations for future studies. Biomark Med. 4(5), 6, 83–99 (2010). 10.2217/bmm.10.90 20945981

[b21] Barbour R. . Red blood cells are the major source of alpha-synuclein in blood. Neurodegener Dis. 5(2), 55–9 (2008). 18182779 10.1159/000112832

[b22] Vivacqua G. . Abnormal Salivary Total and Oligomeric Alpha-Synuclein in Parkinson’s Disease. PLos One. 11(3), e0151156 (2016). 27011009 10.1371/journal.pone.0151156 PMC4807094

[b23] Shi M. . Plasma exosomal α-synuclein is likely CNS-derived and increased in Parkinson’s disease. Acta Neuropathol. 128(5), 639–50 (2014). 24997849 10.1007/s00401-014-1314-y PMC4201967

[b24] Ogawa Y. .Proteomic analysis of two types of exosomes in human whole saliva. Biol Pharm Bull. 34(1), 13–23 (2011). 21212511 10.1248/bpb.34.13

[b25] Grey M. . Acceleration of α-synuclein aggregation by exosomes. J Biol Chem. 30, 290(5), 2969–82 (2015). 25425650 10.1074/jbc.M114.585703 PMC4317028

[b26] Stewart T. . Cheek cell-derived α-synuclein and DJ-1 do not differentiate Parkinson’s disease from control. Neurobiol Aging. 35(2), 418–20 (2014). 24041968 10.1016/j.neurobiolaging.2013.08.008 PMC3844543

[b27] Al-Nimer M. S., Mshatat S. F. & Abdulla H. I. Saliva a-Synuclein and A High Extinction Coefficient Protein: a novel approach in assessment biomarkers of Parkinson’s disease. N Am. J Med Sci. 6, 633–637 (2014). 10.4103/1947-2714.147980 PMC4290052 25599051

[b28] van Dijk K. D. . Reduced α-synuclein levels in cerebrospinal fluid in Parkinson’s disease are unrelated to clinical and imaging measures of disease severity. Eur J Neurol. 21(3), 388–94 (2014). 23631635 10.1111/ene.12176

[b29] Koehler N. K. .Alpha-synuclein levels in blood plasma decline with healthy aging. PLos One. 6, 10(4), e0123444 (2015). 25844871 10.1371/journal.pone.0123444 PMC4386828

[b30] Tokuda T. . Detection of elevated levels of α-synuclein oligomers in CSF from patients with Parkinson’s disease. Neurology. 16, 75(20), 1766–72 (2010). 20962290 10.1212/WNL.0b013e3181fd613b

[b31] Stuendl A. . Induction of α-synuclein aggregate formation by CSF exosomes from patients with Parkinson’s disease and dementia with Lewy bodies. Brain. 139, (Pt 2), 481–94 (2016). 26647156 10.1093/brain/awv346 PMC4805087

