# Supplementary material for: Salivary total α-synuclein, oligomeric α-synuclein and SNCA variants in Parkinson’s disease patients
Source: Sci Rep. 2016 Jun 23;6:28143. doi: 10.1038/srep28143 (PMC4917865; doi:10.1038/srep28143)
Supplement: Supplementary Information [file srep28143-s1.pdf]

Salivary total α-synuclein, oligomeric α-synuclein and *SNCA* variants in Parkinson's disease patients

Wenyan Kang[1,2†], Wei Chen[1†], Qiong Yang[1], Lina Zhang[3], Linyuan Zhang[1], Xiaoying Wang[1], Fangyi Dong[1],Yang Zhao[1], Shuai Chen[1], Thomas J. Quinn[4], Jing Zhang[5], Shengdi Chen[1,§], Jun Liu[1,§]

Suppl. Figure 1. Salivary total α-synuclein and PD

(A) Salivary α-synuclein levels were measured in normal controls (NC) and PD samples by Luminex. Salivary total α-synuclein levels did not correlate with gender both in individual PD and control groups (p=0.71, p=0.32 when normalized in PD group; p=0.75, p=0.67 when normalized in the NC group). **(B)** Statistical analysis of salivary total α-synuclein in Hoehn and Yahr's stages of PD, with no significant difference being achieved (p>0.05). **(C)** Salivary total α-synuclein didn't correlated with disease severity as measured by UPDRS motor scores (r=0.11, p=0.14; r=0.087, p=0.22 when normalized) using linear regression analysis with spearman's correlation, and also checked by partial correlation to controlling for possible confounding variables-age and gender. **(D)** There is no significant difference among the different drug treatment groups (p=0.83, p=0.75 when normalized). **(E)** Using linear regression analysis with spearman's correlation, levels of total α-synuclein in saliva didn't correlated with disease duration in PD group (r=0.0011 p=0.99, r=-0.029, p=0.68 when normalized), no in controls neither (p>0.05, p>0.05 when normalized). Error bars represent mean ± SEM.

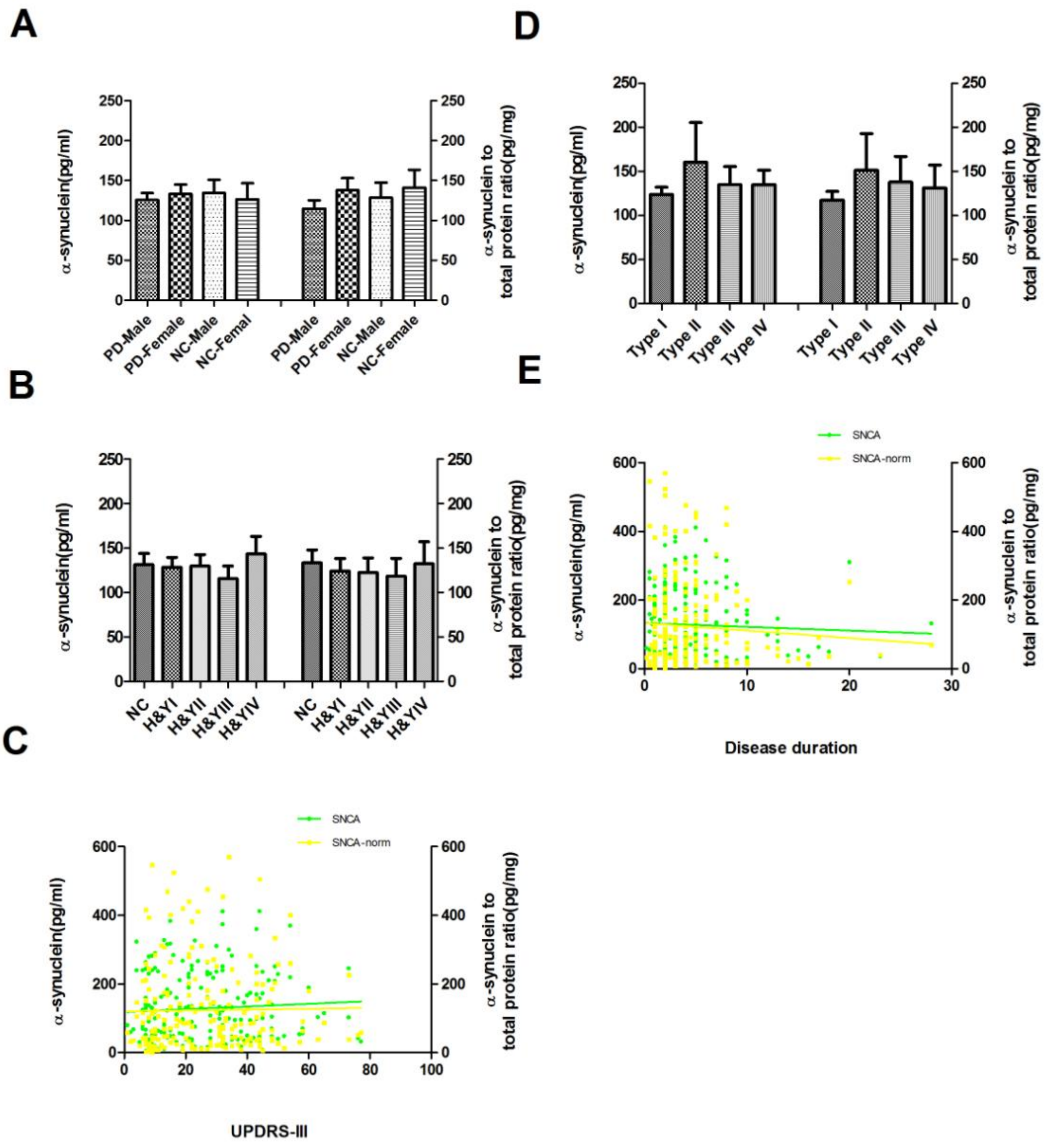

Suppl. Figure 2. Salivary α-synuclein and plasma α-synuclein

The levels of α-synuclein in salivary and plasma were measured in 15 normal controls (NC) and 12 PD samples by Luminex.Using linear regression analysis with spearman's correlation, levels of total α-synuclein in saliva didn't correlated with plasma α-synuclein in normal controls (**A-B**,p>0.05), no neither inPD group (**C-D**,*p*>0.05).

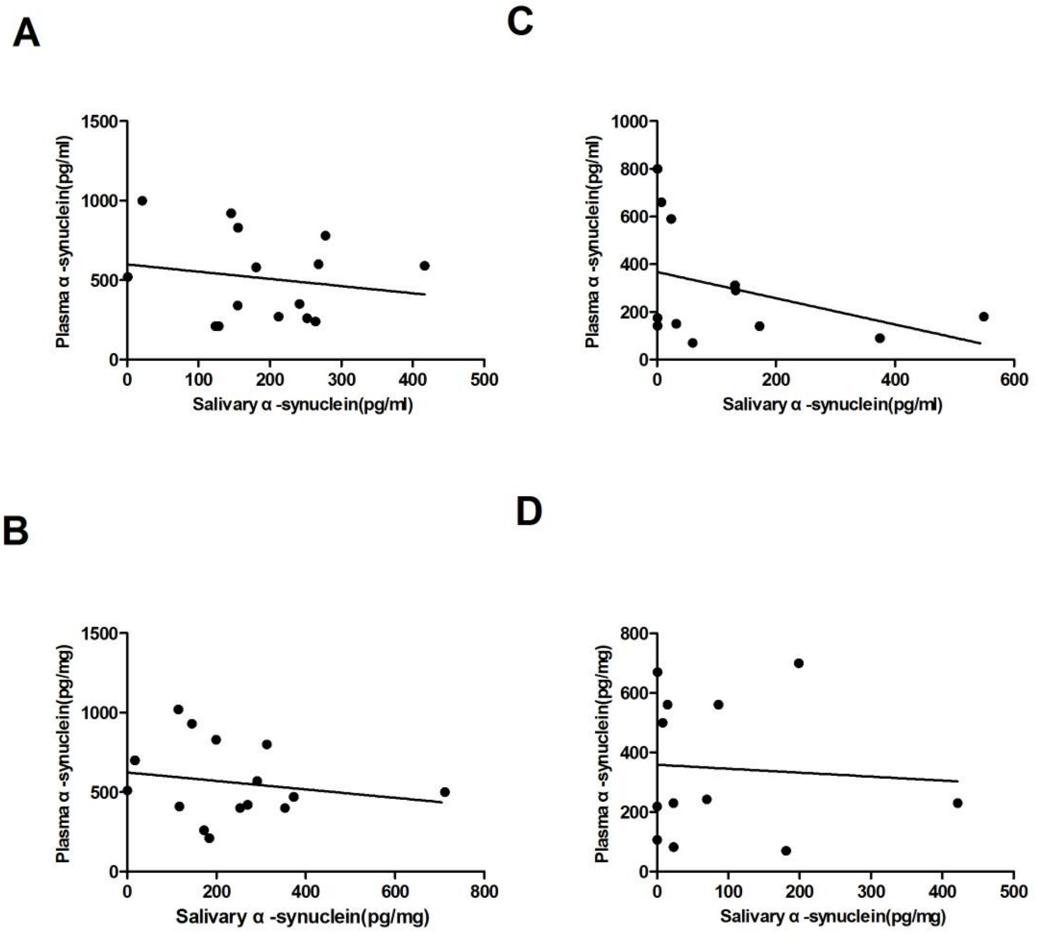

A
Plasma α -synuclein(pg/ml)
1500
1000
500
0
0
100
200
300
400
500
Salivary α -synuclein(pg/ml)
C
Plasma α -synuclein(pg/ml)
1000
800
600
400
200
0
0
200
400
600
Salivary α -synuclein(pg/ml)
B
Plasma α -synuclein(pg/mg)
1500
1000
500
0
0
200
400
600
800
Salivary α -synuclein(pg/mg)
D
Plasma α -synuclein(pg/mg)
800
600
400
200
0
0
100
200
300
400
500
Salivary α -synuclein(pg/mg)
